# A Stroke of Bad Luck: An Autobiographical Case Report

**DOI:** 10.7759/cureus.44788

**Published:** 2023-09-06

**Authors:** Philip R Cohen

**Affiliations:** 1 Dermatology, University of California Davis Medical Center, Sacramento, USA

**Keywords:** stroke, magnetic resonance imaging, flutter, fibrillation, dysarthria, cortex, computer axial tomography, cerebrovascular, atrial, accident

## Abstract

Strokes are a common cause of death. Cardiovascular disease, including atrial fibrillation and atrial flutter, is a frequent cause of ischemic strokes. A 64-year-old man developed isolated dysarthria without any other neurologic manifestations as the presentation of an ischemic stroke resulting from occlusion to the middle cerebral artery and affecting the cortex supplied by the artery. He was discovered to be in atrial flutter which was determined to be the likely etiology of his stroke. He was hospitalized and anticoagulated with heparin; as an outpatient, his anticoagulation was maintained with the direct oral anticoagulant apixaban. Amiodarone was required to medically convert him to normal sinus rhythm; he has typical atrial flutter and is going to be evaluated for atrial flutter ablation. His dysarthria began to improve within 24 hours after he experienced the stroke; after five weeks of speech therapy his ability to talk continues to progressively improve and the residual deficits in his speech continue to resolve. Anticoagulation is required for stroke prevention in individuals with atrial fibrillation and atrial flutter. Warfarin, a vitamin K antagonist, is usually used for individuals with valvular atrial fibrillation. Direct oral anticoagulants have fewer bleeding complications and are usually recommended for nonvalvular atrial fibrillation; they include the direct thrombin inhibitor dabigatran or a factor ten a (Xa) inhibitor such as either apixaban, edoxaban, or rivaroxaban. Dysarthria is a common manifestation in stroke patients. Albeit, it is less common, isolated dysarthria without any other neurologic sequellae may be associated with stroke. Interventions encouraged by speech pathologists to enhance the resolution of post-stroke dysarthria include speaking louder to amplify the voice and exaggerating the movements of the mouth when speaking.

## Introduction

A stroke is potentially fatal. It results from an ischemic or hemorrhagic injury to the brain. Cardiovascular disease can cause a stroke; either atrial fibrillation or atrial flutter can be associated with the development of a cardioembolic ischemic stroke [1-4].

The management of an ischemic stroke typically involves anticoagulation of the patient. A vitamin K antagonist, such as warfarin, was the treatment for most patients prior to 2009 and is still the preferred agent for stroke prevention in individuals with valvular - which is associated with mitral valve disease - atrial fibrillation or a prosthetic heart valve. In contrast, newer direct oral anticoagulants have fewer bleeding complications; these include the direct thrombin inhibitor dabigatran and the factor ten a (Xa) inhibitors such as apixaban, edoxaban, and rivaroxaban [5-8].

Dysarthria is a complication that is associated with stroke; albeit, less common, isolated dysarthria with no other neurologic symptoms can occur. In ischemic stroke patients with supratentorial infarctions, the territory of the motor cortex and the corona radiata that receives its blood supply from the right middle cerebral artery is the most commonly affected [9-12]. The clinic history, evaluation, and management of a man who had an ischemic stroke with subsequent pure dysarthria, most likely secondary to atrial flutter, is described.

## Case presentation

I was extremely exhausted. I spoke to one of my children at 11:30 p.m. It was now 1:00 a.m. and I was getting ready to go upstairs to bed. I had let the dogs out into the backyard. I was attempting to call them back into the house; I realized that I could not say the dogs’ names. They came back into the house on their own. I carried a clean load of laundry up the stairs. I realized that something was wrong, but I was just too tired and I thought it would resolve before I awoke the following morning.

Earlier that evening, I had jogged with my dogs for five miles; the ambient temperature was about 84 degrees Fahrenheit. I returned very dehydrated after completing the jog and drank at least 60 ounces of carbonated seltzer during the next few hours. I participated in a Zoom conference an hour after I finished jogging. I had no headache.

The following morning I awoke at 7:30 a.m. and realized that I still could not say the dogs' names and I had slurred speech. I looked in the mirror and did not note any asymmetry on my face; I could smile and see all of my teeth. I shaved, brushed my teeth, dressed, and accepted the realization that I had a stroke. In my nearly non-interpretable speech, I anxiously told my son to take me to a local community hospital. As I walked into the emergency room, I told the person at the entrance desk that I had a stroke and inquired whether I should be considered for intervention tissue plasminogen activator (TPA). Unfortunately, it was beyond the four-and-a-half-hour limit for this intervention to be appropriate.

The hospital emergency department had an efficient established stroke protocol. Within less than 15 minutes I was being taken for a non-contrast computerized axial tomography (CAT) scan of my head and neck without contrast. My diagnosis had been correct, a standard multidetector CAT scan showed an early right frontal lobe infarct with early cytotoxic edema; there was no large vessel occlusion including the following arteries: brachiocephalic, vertebral, and carotid.

A telemedicine visit from the onsite hospital neurologist followed shortly thereafter. I was to be anticoagulated with heparin. My next visitor was the speech pathologist, who had the responsibility of doing the dysphagia assessment; she confirmed that I had dysarthria and was capable of swallowing.

**Figure 1:**
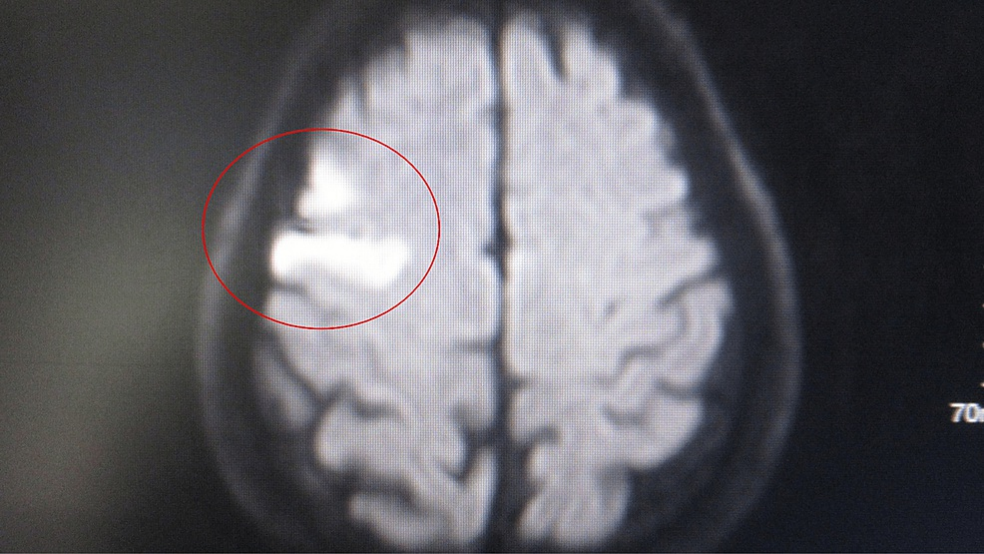
A 1.5 Tesla magnetic resonance imaging of the brain was performed without intravenous contrast. The magnetic resonance imaging (1.5 Tesla) showed an area consistent with acute infarct that appears white (within a red oval). An area of restricted diffusion in the right middle cerebral artery territory with corresponding abnormality and apparent diffusion coefficient map consistent with acute infarct. This is small in size involving the lateral right frontal cortex. The transverse relaxation time (T2) and fluid-attenuated inversion recovery (FLAIR) hyperintensity within the area of infarct was consistent with an infarct of greater than 12 to 24 hours in age. There was no evidence of hemorrhagic transformation.

A 1.5 Tesla magnetic resonance imaging was eventually performed without intravenous contrast (Figure 1). A small acute right middle cerebral artery territory infarct without hemorrhagic transformation. The infarct was similar in size and location to that noted on the CAT scan.

**Figure 2:**
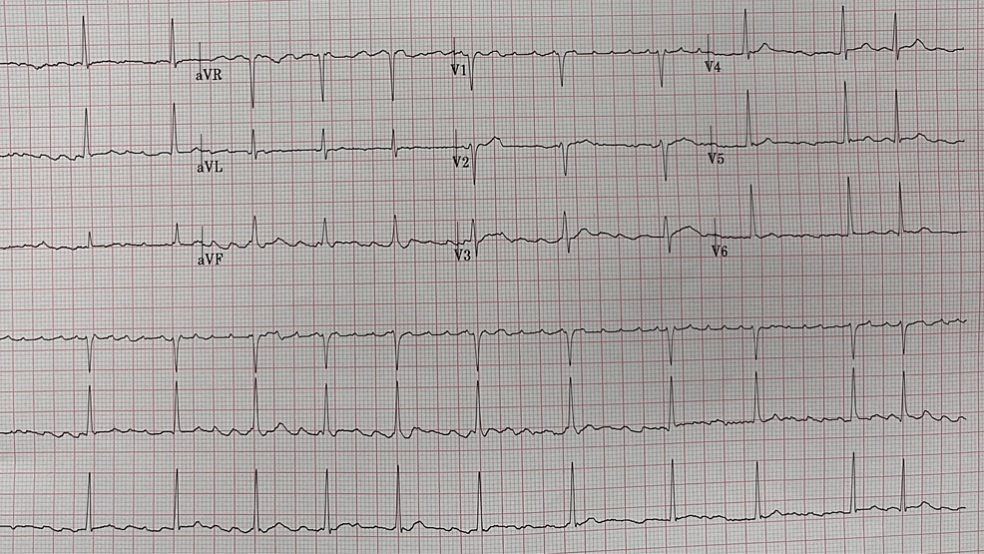
The electrocardiogram shows atrial flutter with variable atrioventricular block. The ventricular rate is 73 beats per minute.

**Figure 3:**
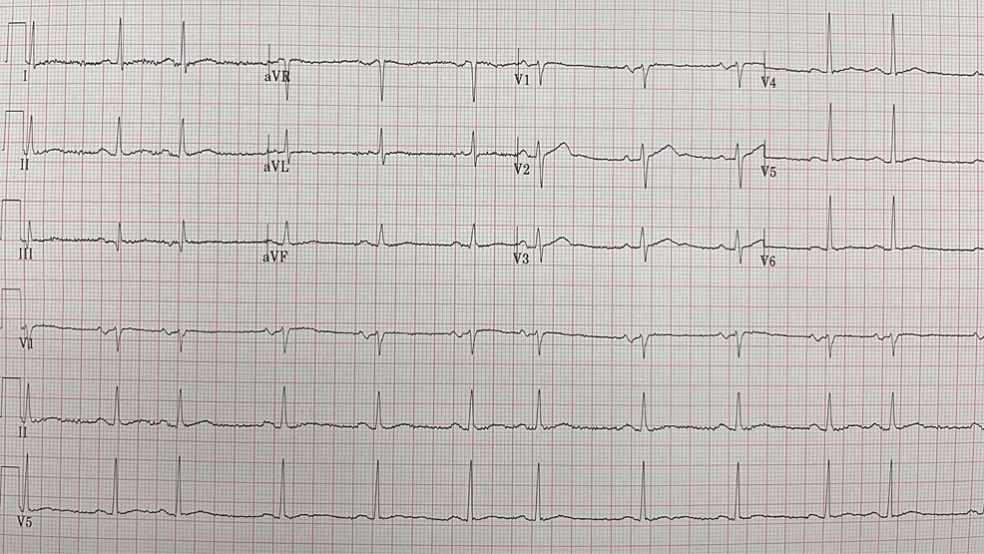
The electrocardiogram shows sinus rhythm with marked sinus arrhythmia. Sinus rhythm is observed and there is also marked sinus arrhythmia; the ventricular rate is 67 beats per minute.

On presentation to the emergency department, my blood pressure was elevated 175/113 millimeters of mercury (systolic/diastolic), my heart rate was 71 beats per minute, my respiratory rate was 20 breaths per minute, and my oxygen saturation was 96 percent and my temperature was 97.9 degrees Fahrenheit. An electrocardiogram demonstrated that I was in atrial flutter with a variable atrioventricular block (Figure 2); the ventricular rate was 73 beats per minute; this was likely the etiology of my stroke. I had previously experienced a single episode of postoperative atrial fibrillation after back surgery nearly six years earlier, which responded to a single 10 mg dose of nifedipine without recurrence. Prior to leaving the emergency department, I had spontaneously converted into sinus rhythm (Figure 3).

A comprehensive neurology examination revealed no other findings. There were no motor deficits, no weakness, and no loss of sensation. Facial palsy was absent. With great effort, my speech was slurred and almost uninterpretable.

Laboratory studies were performed. Serial troponin levels were negative. My lipid profile was abnormal: the cholesterol (252 mg/dL; normal, less than 200 mg/dL) was elevated, the triglycerides (177 mg/dL; normal, less than 150 mg/dL) were elevated, and the low-density lipoprotein cholesterol (180 mg/dL; normal less than 100 mg/dL) was also elevated.

A two-dimensional transthoracic echocardiogram with color flow and Doppler bubble was performed; a saline contrast injection was performed to assess for cardiac shunting and demonstrated a negative bubble study. The left ventricle was normal in size and there was mild to moderate concentric left ventricular hypertrophy; the ejection fraction was estimated to be 65 to 70 percent. The right ventricle was mildly dilated and the right ventricular systolic function was normal. The aortic valve was sclerotic and there was mild tricuspid regurgitation. In summary, no obvious cardiac source of embolus was noted.

The remainder of my three-day hospital stay was unremarkable; my physician's wife was at my side continuously. The speech pathologist visited each day. My speech had progressively improved; however, it was not normal when I was discharged home. I was started on apixaban for anticoagulation, ezetimibe for hyperlipidemia, and amlodipine for hypertension.

After being discharged home, I followed up with several clinicians. The cardiologist was responsible for following up on my arrhythmia, my hypertension, and my anticoagulation. At my follow-up visit, five days later, I was back in atrial flutter. After ensuring that I was adequately anticoagulated after two weeks of apixaban, amiodarone was started in order to attempt to medically convert me into normal sinus rhythm, and metoprolol extended-release was also added. I spontaneously converted into a normal sinus rhythm after 10 days. The daily dose of amiodarone was reduced after three additional weeks. Since I had typical atrial flutter, evaluation for atrial flutter ablation is being scheduled.

I also saw a neurologist who was monitoring the reports from the speech pathologist. Each week I worked with the speech pathologist. My vocalization is significantly improved after five weeks and I am still continuing to work toward improving my speech.

## Discussion

Strokes are either ischemic (approximately 85 to 87 percent) or hemorrhagic (approximately 13 to 15 percent). An ischemic stroke results when there is a clot in a vessel. A hemorrhagic stroke is classified as intracranial when a blood vessel has ruptured in the brain or subarachnoid if the ruptured vessel is within the subarachnoid space (accounting for approximately 3 percent of strokes). In the United States of America, ischemic strokes are the fifth most common cause of death [1-3].

Elderly individuals, over the age of 65 years, are at increased risk of developing an ischemic stroke. Risk factors associated with the development of an ischemic stroke include atrial fibrillation, diabetes mellitus, hypertension, and hypercholesterolemia. Modification of an individual’s lifestyle can be an effective measure to prevent ischemic stroke; potential interventions include cessation of smoking, diet modification, eliminating or markedly decreasing alcohol consumption, and increased exercise [4].

The CHA_2_DS_2_-VASc score is a risk assessment to determine stroke risk for patients with atrial fibrillation. The acronym is based on the following factors: C, congestive heart failure (one point); H, hypertension (one point); A_2_, age (greater or equal to 75 years, two points); D, diabetes (one point); S_2_, prior stroke or transient ischemic attack or thromboembolism (two points); V, vascular disease (peripheral artery disease, myocardial infarction, or aortic plaque, one point); A, age (65 to 74 years, one point); and Sc, sex category (female sex, one point). The possible score ranges from zero to nine; age is only awarded either no, one, or two points. A score of zero is interpreted that there is no added stroke risk; stroke prevention using oral anticoagulant therapy should be considered for men with a score of one and is recommended for women with a score of two [5].

The management options for an acute ischemic stroke have improved. Alteplase (tissue plasminogen activator) is only suitable to be considered for treatment if initiated within four and a half hours after the onset of symptoms. New drugs for thrombolysis are being considered, including tenecteplase - a variant of alteplase - which has a longer half-life and higher fibrin selectivity [2,3].

In addition to intravenous thrombolysis, mechanical endovascular thrombectomy is a potential acute intervention. Also, selective cerebral hypothermia is also being considered for its neuroprotective benefits. Pharmacotherapies that are not only neuroprotective but also neuroreparative are being combined with mechanical thrombectomy. These include nerinetide (a synthetic eicosapeptide that functions by disrupting the interactions between N-methyl-d-aspartate glutamate receptors and excitotoxic signaling proteins by interacting with postsynaptic density protein 95), magnesium sulfate, apTOLL 90 (a toll-like receptor 4 antagonist that functions by interfering with the innate immune response), and 3K3A-activated protein C (APC); 3K3-APC is a genetically engineered variant of human APC that has an anticoagulant pathway and a cytoprotective pathway that reduces neurologic injury [2,3].

Atrial fibrillation affects approximately 2 percent of the population. The risk of ischemic stroke resulting from thromboembolism is increased by three-fold to five-fold by atrial fibrillation or atrial flutter. Oral anticoagulants have decreased the risk of atrial fibrillation-associated stroke by approximately 66 percent [6,8].

Stroke prevention typically involves anticoagulation for individuals who have experienced a stroke and are in atrial fibrillation or atrial flutter. This also includes patients who have a history of paroxysmal atrial fibrillation (which usually resolves spontaneously within seven days) or persistent atrial fibrillation (which lasts more than seven days). Anticoagulants for stroke prevention for individuals who have atrial fibrillation or atrial flutter include not only vitamin K antagonists such as warfarin but also direct oral anticoagulants which have a lower risk of bleeding complications [5,6].

After I experienced my ischemic stroke, I was in atrial flutter. However, I had a prior history of postoperative atrial fibrillation after back surgery. I had been assessed by a cardiologist after my hospitalization and was observed to be in normal sinus rhythm [13]. My current atrial flutter was idiopathic and asymptomatic; I am unaware of when my atrial flutter began. Since my atrial flutter is typical, I am going to be evaluated for atrial flutter ablation [5-8].

Prior to 2009, warfarin was used for valvular atrial fibrillation in which the atrial fibrillation coexisted with either mitral stenosis or a mechanic mitral prosthesis. It was also used in patients with nonvalvular atrial fibrillation in which the atrial fibrillation occurred in the absence of a diseased mitral valve. Currently, vitamin K antagonists are primarily recommended for patients who have prosthetic heart valves and valvular atrial fibrillation [5-8].

Warfarin is a vitamin K antagonist (Table 1) [5-8]. Frequent monitoring of the international normalized ratio (INR) is required after starting treatment with warfarin. The therapeutic range for warfarin is very narrow; a therapeutic INR is between two and three [5-8].

**Table 1:** Anticoagulants for stroke prevention in atrial fibrillation DTI, direct thrombin inhibitor; FXa, factor 10 a; FXaI, factor 10a inhibitor; INR, international normalized ratio; 4F-PCC, four-factor prothrombin complex concentrate; VKA, vitamin K antagonist ^a^Alcohol should be limited or avoided on these medications. For patients taking warfarin no more than two drinks for men or one drink for women. For patients taking factor 10a inhibitors, a maximum of less than two standard drinks per day is the suggested upper limit. One drink is 12 ounces of 5 percent alcohol, five ounces (142 milliliters) of 12 percent wine; and 1.5 ounces or 43 milliliters of 40 percent liquor or spirits. ^b^Dark green leafy vegetables include asparagus, broccoli, Brussels sprouts, cauliflower, green onions, kale, paisley, and spinach. These vegetables are high in vitamin K and can alter the INR. Dark green leafy vegetables do not need to be completely avoided, but their intake needs to be consistent. ^c^4-PCC contains highly concentrated coagulation factors: II, VII, IX, and X. ^d^Oral (preferred) or intravenous (over 10 to 20 minutes) vitamin K 2.5 to 10 milligrams. ^e^A monoclonal antibody with an affinity for dabigatran, which frees thrombin from its binding to the drug. ^f^A recombinant form of inactivated FXa; the drug binds to the anticoagulant and thereby frees FXa.

Drug	Mechanism of action	Foods to avoid^a^	Reversal agent
Warfarin	VKA	Variable consumption of dark green leafy vegetables^b^, grapefruit and grapefruit juice	4F-PPC^c^,Vitamin K^d^
Dabigatran	DTI	Grapefruit and grapefruit juice, limes, marmalades, and pomelos	Idarucizumab^e^
Apixaban	FXaI	Grapefruit and grapefruit juice, limes, marmalades, and pomelos	Andexanet alfa^f^
Edoxaban	FXaI	Grapefruit and grapefruit juice, limes, marmalades, and pomelos	None
Rivaroxaban	FXaI	Grapefruit and grapefruit juice, limes, marmalades, and pomelos	Andexanet alfa^f^

Warfarin is susceptible to many drug-drug interactions. The metabolism of the drug is influenced by fever, inflammation, kidney disease, and liver disease. Patients who are taking warfarin need to maintain a consistent intake of dark green leafy vegetables which are foods that are high in vitamin K. In addition, they cannot drink grapefruit juice and they need to restrict their alcohol intake to less than two drinks per day [5-8].

If reversal of anticoagulation is required for a person taking warfarin, they need to receive either oral or intravenous vitamin K. In addition, they are also treated with four-factor prothrombin complex concentrate (4F-PCC) [5-8].

There are two classes of direct oral anticoagulants (Table 1) [5-8]; they do not require that the patient regularly monitor their INR. They include the direct thrombin inhibitor, dabigatran. In addition, they also include the factor Xa inhibitors apixaban, edoxaban, and rivaroxaban [5-8].

Dabigatran is not recommended for patients with low creatinine clearances (that are less than 15 milliliters per minute) or who are on dialysis; patients who take this medication should not only avoid grapefruit but also grapefruit juice. For life-threatening bleeding, intravenous idarucizumab can be given. This is a monoclonal antibody that binds to dabigatran, which frees thrombin from the anticoagulant [5-8].

Apixaban can be used for patients with end-stage renal disease or who are on dialysis. It should not be used in patients with severe liver impairment. The daily dose is lower in patients older than 80 years of age, 60 kilograms or thinner, and whose serum creatinine is greater than 1.5 milligrams per deciliter [5-8].

Edoxaban and rivaroxaban are both to be avoided in patients with low creatinine clearances or high creatinine clearance (greater than 95 milliliters per minute); for patients with lower creatinine clearances (between 15 and 50 milliliters per minute), a reduced daily dose is given. Both of these drugs should be avoided in patients with moderate to severe liver impairment. Also, both drugs should be avoided in morbidly obese patients who have a body mass index (BMI) of 40 kilograms per square meter or greater [5-8].

Direct oral anticoagulants do not have the same drug-food interactions as warfarin with vegetables. However, patients who have been anticoagulated with a factor Xa inhibitor need to avoid grapefruit and grapefruit juice. They also should limit their alcohol intake to less than two standard drinks per day [5-8].

Andexanet alfa can be used to reverse apixaban and rivaroxaban. It binds to the factor Xa inhibitor and increases thrombin generation. There is no specific agent that can reverse edoxaban [5-8].

Dysarthria can develop in individuals who have had a stroke or a traumatic brain injury; it has also been observed in patients with diseases of the central nervous system. An infarct or hemorrhage of various isolated intracranial or subcranial locations has been associated with stroke-associated dysarthria. Dysarthria is a motor speech disorder and its features include weakness, incoordination, and slowness of the speech musculature with difficulty in forming and pronouncing words [10-12].

The incidence of dysarthria ranges from 20 to 42 percent of stroke patients; in particular, following an individual’s first stroke, about two-thirds of the patients experience dysarthria. Unfortunately, in fewer than half of these individuals, the dysarthria has resolved after three months; after six months residual dysarthria-related impairment only decreases to 27 percent of patients [10-12].

In a retrospective study that evaluated 1150 post-stroke patients, 420 of the patients had dysarthria resulting from an isolated lesion. Nearly 30 percent (270 of 909 patients with ischemic strokes) had supratentorial strokes. The most common location of the infarctions was located in the middle cerebral artery territory including the motor cortex and the corona radiata, similar to my stroke [11].

The retrospective study evaluated 24 studies. There were eight studies that included patients with pure dysarthria, who had no other neurological symptoms; there were a total of 46 patients, similar to my presentation. Twenty of these patients had isolated infarctions and presented with small-sized infarcts. The corona radiata was the most common location [11].

The speech pathologist has an integral role in the assessment and rehabilitation of individuals who have post-stroke dysarthria. The initial evaluation includes an assessment of the clinical presentation of the patient, the intelligibility of the individual, and the psychosocial impact that the dysarthria has had upon the person. In addition, acoustic parameters may be assessed (Table 2) [9,12].

**Table 2:** Acoustic assessment of patients with dysarthria due to stroke DDK, diadochokinetic syllable rate; MCA, maximal counting ability; MPT, maximal phonation time; ROS, rate of speech; VSA, vowel space area

Test	Comment
DDK	This test measures how quickly an individual can accurately repeat a series of rapid, alternating sounds called tokens. The tokens contain one, two, or three syllables. This test is useful for detecting dysarthria after stroke and motor speech control.
MCA	The longest time that a patient can pronounce the /i/ (letter “E”) without a break after a deep inspiration.
MPT	This is determined, after a maximum inspiration, to be the longest time that an individual and pronounce /a/ at a comfortable amplitude.
ROS	The number of words per minute that a person can pronounce.
S/Z ratio	This is the ratio of the longest times that an individual can pronounce "s" versus "z" after a deep inspiration.
VSA	This refers to the two-dimensional area bounded by line connecting the first and second formant frequencies: formant patterns F1 and F2.

There are several interventions that can be initiated to enhance the resolution of post-stroke dysarthria. These include amplifying the voice when attempting to speak. In addition, exaggerating the motions of the mouth when talking. These are some of the techniques that I have found to be most helpful. My dysarthria is significantly improved; however, I must continually remember to continue to talk louder so that what I am saying is heard more clearly by those to whom I am speaking [10].

## Conclusions

Cardiovascular disease is a common etiology of stroke. A man is reported who presented with an atrial flutter-associated ischemic stroke that presented only with dysarthria and no other neurologic manifestations. Anticoagulation was initiated in the hospital with intravenous heparin and continued after discharge with oral apixaban; oral treatment with amiodarone converted his typical atrial flutter to normal sinus rhythm and he will be evaluated for atrial flutter ablation. His post-stroke dysarthria improved within 24 hours and his residual speech deficits continued to progressively resolve after five weeks of speech therapy. Stroke prevention in individuals with atrial fibrillation and atrial flutter requires anticoagulation with either a vitamin K antagonist, warfarin, or a direct oral anticoagulant; the latter includes the direct thrombin inhibitor dabigatran or a factor Xa inhibitor such as either apixaban, edoxaban, or rivaroxaban. Post-stroke dysarthria is a common manifestation. Similar to the man described in this report, isolated dysarthria without any accompanying neurologic symptoms is not common. Some of the techniques that can be incorporated to hasten the improvement of speech in patients with stroke-related dysarthria include amplifying the volume of the voice by speaking louder and exaggerating the mouth’s natural movements when speaking.
